# The Effect of TNF-α Inhibitors on Nail Psoriasis and Psoriatic Arthritis—Real-World Data from Dermatology Practice

**DOI:** 10.3390/jpm11111083

**Published:** 2021-10-25

**Authors:** Georgios Kokolakis, Robert Sabat, Imma Fischer, Susana Gomis-Kleindienst, Björn Fritz, Gerd-Rüdiger Burmester, Kamran Ghoreschi, Sarah Ohrndorf

**Affiliations:** 1 Psoriasis Research and Treatment Center, Charité—Universitätsmedizin Berlin, Corporate Member of Freie Universität Berlin and Humboldt-Universität zu Berlin, 10117 Berlin, Germany; robert.sabat@charite.de; 2 Department of Dermatology, Venereology and Allergology, Charité—Universitätsmedizin Berlin, Corporate Member of Freie Universität Berlin and Humboldt-Universität zu Berlin, 10117 Berlin, Germany; kamran.ghoreschi@charite.de; 3 Interdisciplinary Group of Molecular Immunopathology, Dermatology/Medical Immunology, Charité—Universitätsmedizin Berlin, 10117 Berlin, Germany; 4 Biostatistik—Tübingen, 72070 Tuebingen, Germany; Imma.Fischer@biostatistik-tuebingen.de; 5 AbbVie Deutschland GmbH & Co. KG, 65189 Wiesbaden, Germany; susana.gomis-kleindienst@abbvie.com (S.G.-K.); bjoern.fritz@abbvie.com (B.F.); 6 Department of Rheumatology and Clinical Immunology, Charité—Universitätsmedizin Berlin, Corporate Member of Freie Universität Berlin and Humboldt-Universität zu Berlin, 10117 Berlin, Germany; gerd.burmester@charite.de (G.-R.B.); sarah.ohrndorf@charite.de (S.O.)

**Keywords:** psoriasis, nail psoriasis, psoriatic arthritis, TNFα-inhibitor, DLQI, HAQ, etanercept, adalimumab, infliximab

## Abstract

Patients with psoriatic arthritis (PsA) often develop joint symptoms years after their initial diagnosis of psoriasis disease; therefore, dermatologists should test for and detect PsA early. In this study, we focused on patients with psoriasis with both nail and joint disease being treated with tumor necrosis factor-α inhibitors by dermatologists. We performed a noninterventional, prospective, multicenter, and open-label study to evaluate the effectiveness of adalimumab, etanercept, or infliximab over 24 months of continuous therapy in patients with moderate to severe plaque-type psoriasis (Pso) and PsA. Disease assessments with the Psoriasis Area and Severity Index, Nail Psoriasis Severity Index (NAPSI), joint assessment, Dermatology Life Quality Index (DLQI), and Health Assessment Questionnaire (HAQ) instruments were performed every 3 months for the first year and twice annually thereafter. The cohort included 100 patients with Pso, nail psoriasis, and PsA. A significant reduction of NAPSI was observed 3 months after therapy initiation compared with the baseline (mean ± SD, 22.9 ± 17.8 vs. 33.8 ± 21.4; *p* < 0.001). Similarly, the mean ± SD number of both tender and swollen joints decreased significantly within the first 3 months of treatment, from 10.8 ± 11.5 to 6.4 ± 10.3 (*p* < 0.001) and from 6.4 ± 9.5 to 3.1 ± 7.2 (*p* < 0.001), respectively. Additionally, the distal interphalangeal joint involvement improved throughout the observation time, and DLQI and HAQ scores decreased. Improvements in control of skin, nail, and joint symptoms were seen, as well as in patients’ quality of life and functionality. Dermatologists have an important role not only in PsA diagnosis but also in PsA long-term care.

## 1. Introduction

Psoriasis is a chronic inflammatory disease that affects skin and nails, entheses, and peripheral and axial joints [1]. In most patients with psoriasis, skin disease precedes joint involvement. Approximately 67% of patients with psoriatic arthritis (PsA) develop the arthritis nearly 10–20 years after the onset of the cutaneous symptoms of the disease [2]. Nail lesions occur in more than 80% of patients with PsA compared with nearly 40% of patients without PsA [3]. Although there is a wide range of systemic therapies currently available for plaque-type psoriasis (Pso), treatment of nail psoriasis remains challenging [4,5]. Because nail psoriasis significantly affects patients’ quality of life (QoL), it belongs to the criteria for upgrading psoriasis severity, when the affected skin is not considered moderate to severe in disease (PASI < 10) but the patient’s QoL is impaired (DLQI > 10) [6].

Although Pso is an easy clinical diagnosis based on the presence of well-demarcated erythematous scaly plaques on the predisposed areas, such as elbows, knees, and scalp, diagnosing PsA can be challenging. PsA is a seronegative arthritis with late pathognomonic signs in conventional radiology. Newly developed imaging techniques may be more sensitive for the early diagnosis of PsA [7]. However, to a certain degree, the diagnosis of PsA depends on the presence of diseased skin or nail manifestations on the patient or in the family history, as proposed by the classification criteria for PsA (CASPAR) [8].

Considering the usual course of PsA following the skin manifestations, dermatologists should routinely monitor their patients with psoriasis for joint involvement and early diagnosis of PsA. Dermatologists should simultaneously introduce the appropriate therapy to rapidly improve and control skin and joint symptoms. Depending on the phenotype of psoriasis, the presence of inflammatory signs of joint involvement, comorbidities, imaging, laboratory tests, and screening questionnaires as consensus on the management of PsA in a dermatology setting could be established. Morning joint stiffness, as well as tender and swollen joints, associated with psoriasis are major conditions for suspecting PsA; therefore, close interdisciplinary collaboration or referral to rheumatologists should be considered [9,10].

In addition to nail psoriasis, especially nail dystrophy and pitting, severe Pso, psoriasis duration of more than 25 years, obesity, uveitis, smoking, and genetic factors, such as having the HLA-B27 allele, can increase the risk of PsA development. As treatment outcomes after the clinical manifestation of PsA are poor, identifying patients at risk of PsA early, and treating accordingly, would reduce this risk [11].

Because the pathogenetic mechanisms of both Pso and PsA are common and mainly through T-helper cells (Th)-1 or Th-17 driven, the therapeutic strategies are largely overlapping, with minor exceptions [1,12,13,14]. Currently, the development of new therapies for PsA follows that of Pso therapies since the effects of the new substances on the skin are directly visible and easier to objectify [1].

In this noninterventional prospective study, we aimed to describe the cohort of patients with PsA and their therapeutic approach in a dermatology setting, including private practice and state and university hospitals, with a focus on tumor necrosis factor-alpha (TNF-α) inhibitors as the first class of biologics for their treatment of Pso. We specifically focused on patients with nail psoriasis with concomitant PsA, the correlation of nail involvement with PsA, and the therapy response of TNF-α inhibitors. We also addressed the therapy monitoring capabilities from the participating dermatology sites.

## 2. Materials and Methods

### 2.1. Study Design and Participants

This was a noninterventional, prospective, multicenter, and open-label cohort study to evaluate the effectiveness of adalimumab, etanercept, or infliximab on nail psoriasis over 24 months of continuous therapy in patients with moderate to severe Pso with or without PsA. The study was conducted at 27 different sites in Germany with dermatologists in 12 private practices with experience on PsA therapies, 5 local hospitals, and 10 university clinics. The recruitment was performed from March 2008 until November 2009.

Adult patients with moderate to severe psoriasis, as defined by the “rule of ten” (Psoriasis Area and Severity Index [PASI] > 10, body surface area [BSA] > 10, Dermatological Life Quality Index [DLQI] > 10), who were candidates for systemic treatment with the TNF-α inhibitors etanercept, adalimumab, and infliximab according to licensure and had psoriasis of the fingernails were included in this observational study. The total population was divided into two subgroups: those with and those without confirmed PsA. In the current analysis, only patients in the cohort with confirmed PsA were evaluated.

Any other skin diseases that could interfere with the evaluation of psoriasis, forms of psoriasis other than Pso, or the presence of latent or active tuberculosis, hepatitis B or C, or HIV infection were the main exclusion criteria. Patients unable to understand the questionnaires and adhere to the study procedures were not included. 

All participants provided written informed consent before inclusion. The study was approved by the local ethics committee of Charité—Universitätsmedizin Berlin (EA1/236/08) and was conducted according to the principles of the Declaration of Helsinki.

### 2.2. Clinical Skin and Joint Assessments

The severity of psoriatic skin alterations was evaluated using PASI score and BSA. Nail involvement was estimated using the total Nail Psoriasis Severity Index (NAPSI) score of all fingernails, resulting in scores ranging from 0 to 80. The number of 78 tender and 76 swollen joints and morning stiffness (in minutes) were assessed at all visits by the treating dermatologist.

### 2.3. Patient-Reported Outcomes

The impact of psoriasis on a patient’s QoL was estimated by the DLQI. Disability was measured by the Health Assessment Questionnaire-Disability Index (HAQ-DI) with a range of 0 to 30, where higher scores indicate greater disability. HAQ is a self-reported questionnaire containing 20 items in eight domains, which was developed as a comprehensive measure outcome for general disability of patients with a variety of rheumatic diseases, such as PsA, with scores from 0 (no disability) to 3 (severe disability) [15]. Scores of 0–1 represent mild to moderate disability, scores of 1–2 indicate moderate to severe disability, and scores of 2–3 indicate severe to very severe disability [16].

All assessments were performed at baseline, every 3 months for the first year, and then twice annually for the second year of observation. Participating study centers were advised to keep the same examiner for each patient throughout the study to avoid deviation between the raters.

### 2.4. Statistics

Statistical calculations were performed using the Statistical Program for Social Sciences version 25.0 (IBM, Armonk, NY, USA) and Microsoft Excel 2013 (Microsoft Corporation, Redmond, WA, USA). Differences between nonpaired samples were tested using the Mann–Whitney U test. Differences between paired samples were analyzed using the Wilcoxon matched-pairs signed-rank test. Differences between frequencies of men and women in NAPSI improvement groups were tested using a chi-square test. The correlations between NAPSI and distal interphalangeal (DIP) joint involvement were analyzed using a two-tailed Spearman’s rank correlation test.

## 3. Results

### 3.1. Study Population

In total, 100 patients (63% male; mean ± SD age, 49.6 ± 12.4 years) with Pso and nail psoriasis, as well as PsA, with a mean ± SD disease duration of Pso of 20.1 ± 13.7 years and a mean ± SD BMI of 28.7 ± 5.5 kg/m^2^ were included in this cohort study. Demographic and clinical characteristics at baseline are presented in Table 1.

### 3.2. Overall Improvement of Nail and Skin Psoriasis

**Figure 1:**
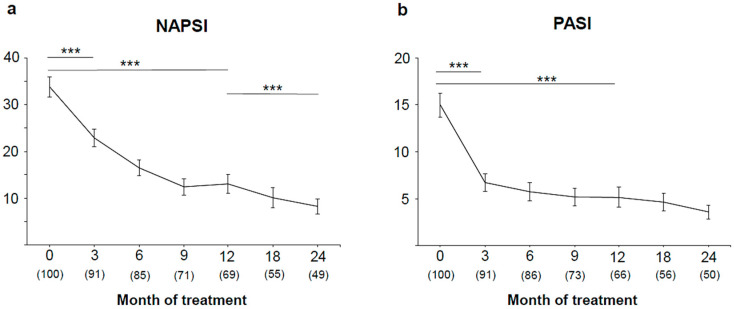
Overall improvement in (**a**) Nail Psoriasis Severity Index (NAPSI) and (**b**) Psoriasis Area and Severity Index (PASI) during therapy. Absolute NAPSI and PASI values (mean ± SEM) and total number of patients per visit are shown. *** *p* < 0.001.

Three months after the initiation of therapy with anti–TNF-α therapy, a significant reduction of absolute NAPSI scores, from 33.8 ± 17.8 (mean ± SD) at baseline to 22.9 ± 21.4 *(p* < 0.001), was observed. NAPSI radically decreased until month 9 (12.4 ± 14.6), where it reached a plateau, and then continued decreasing until the end of the observational period (8.2 ± 11.2) (Figure 1a). PASI rapidly decreased from 15.0 ± 12.5 (mean ± SD) at baseline to 6.7 ± 8.8 (*p* < 0.001) after 3 months of treatment. A further less prominent reduction in PASI was observed until month 24 (3.6 ± 5.2) (Figure 1b).

### 3.3. Impact on PsA

**Figure 2:**
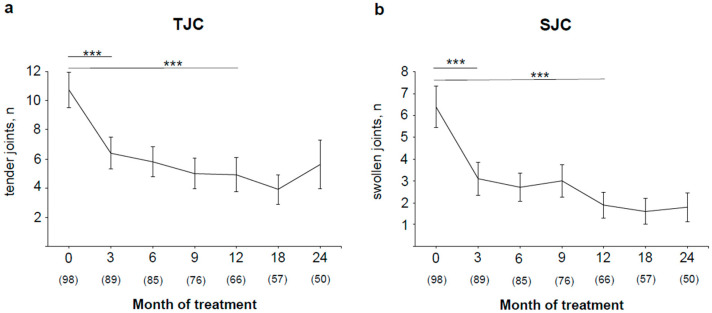
Overall improvement of (**a**) tender joints and (**b**) swollen joints during therapy. Absolute number of tender and swollen joints (mean ± SEM) and total number of patients per visit are shown. *** *p* < 0.001.

The activity of PsA was estimated by the number of tender and swollen joints out of 78-joint status and 76-joint status, respectively. The number of both tender and swollen joints significantly decreased from a mean ± SD of 10.8 ± 11.5 (78-TJC) and 6.4 ± 9.5 (76-SJC) to 6.4 ± 10.3 and 3.1 ± 7.2, respectively, (both *p* < 0.001) during the first 3 months of treatment. A further reduction of tender and swollen joints could be observed until month 12. The number of swollen joints remained low until month 24 (mean ± SD, 1.8 ± 4.7), whereas the mean ± SD number of tender joints presented a nonsignificant tendency to increase after 18 months (3.9 ± 7.7) until month 24 (5.6 ± 11.8) (Figure 2). 

**Figure 3:**
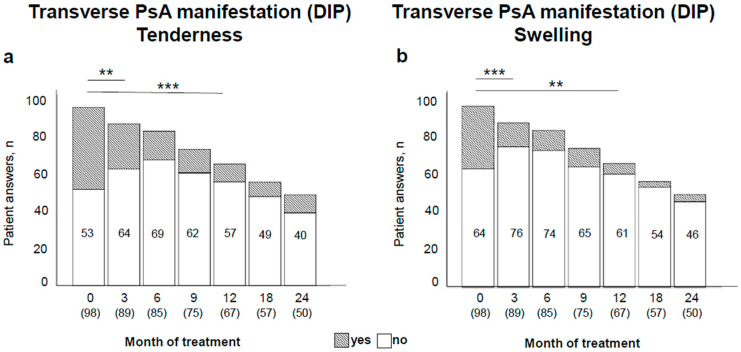
Course of transversal and longitudinal involvement of psoriatic arthritis (PsA) during therapy. In the analysis of transverse joint affection, only the distal interphalangeal (DIP) joints of hand/feet were examined (**a**,**b**). Yes/No refers to the corresponding involvement as shown in the graph. Total number of patients per visit and number of patients without the shown involvement are indicated. ** *p =* 0.001; *** *p* < 0.001.

To further describe the course of PsA under the therapy with TNF-α inhibitors, the DIP joint involvement was considered as a typical clinical manifestation of PsA in association with nail psoriasis. For that, the DIP of hand/feet were especially considered for tenderness or swelling. A significant reduction of the number of swollen joints in the transverse PsA manifestation was determined from 35% at baseline to 8% after 24 months (*p* < 0.001), prominently being observed in the first 3 months (Figure 3).

The overall correlation of tender or swollen DIP joints with NAPSI, either in total or separated for nail bed and nail matrix, across assessment time points was not statistically significant. Similarly, the correlation of PASI, BMI, and disease duration with the course of PsA within the first 3 months was not significant.

### 3.4. Patient-Reported Outcomes

**Figure 4:**
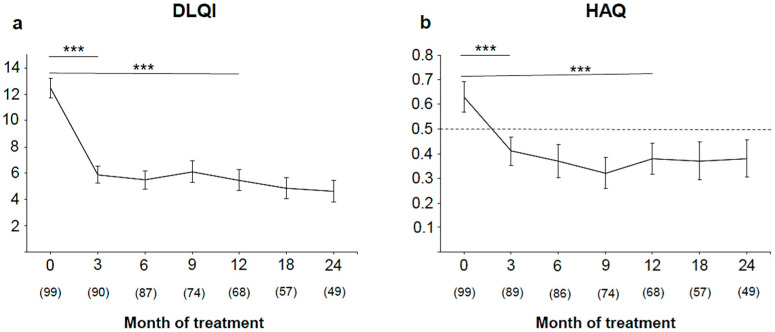
Reduction of (**a**) Dermatological Life Quality Index (DLQI) and (**b**) Health Assessment Questionnaire (HAQ) during therapy. Absolute values of DLQI and HAQ scoring (mean ± SEM) and total number of patients per visit are shown; dashed line represents HAQ 0.5 as functional target for disease control. *** *p* < 0.001.

A substantial impairment of patient QoL was observed before the initiation of treatment with TNF-α inhibitors (mean ± SD DLQI, 12.5 ± 7.4). The QoL significantly improved after 3 months, showing a more than 50% reduction of DLQI compared with baseline (5.9 ± 6.1; *p* < 0.001). Continued treatment led to a further decrease of DLQI until month 24 (mean ± SD DLQI, 4.6 ± 5.7) (Figure 4a). Similarly, patient PsA-associated disability improved 3 months after therapy. Mean ± SD HAQ-DI significantly decreased, from 0.63 ± 0.61 to 0.41 ± 0.55 (*p* < 0.001) and 0.38 ± 0.53 (*p* < 0.001) after 3 and 12 months, respectively, and remained stable and under the cutoff of 0.5 throughout the whole observational period (Figure 4b).

The number of tender joints significantly correlated with disability. The subgroup of patients with HAQ-DI < 0.5 had a significantly lower number of tender joints than the subgroup with HAQ-DI ≥ 0.5 (Spearman’s rho 0.409; Mann–Whitney U test *p* < 0.001).

No significant correlation was observed between nail psoriasis severity (total NAPSI, NAPSI nail bed, and NAPSI nail matrix) and PsA activity (number of tender or swollen joints). Similarly, QoL (DLQI) did not correlate with disability (HAQ-DI).

## 4. Discussion

In this prospective cohort that included patients with nail psoriasis and PsA from daily clinical practice in a dermatology setting, we investigated the effect of therapy with TNF-α inhibitors over an observational time of 24 months. The indication to treat the endpoints primarily focused on dermatologic aspects and additionally comprised involvement of the joints and patients’ disability and QoL.

Dermatologists should evaluate not only the clinical phenotype of psoriasis but also the signs of joint inflammation, comorbidities, imaging, even screening questionnaires for suspicion of PsA, and then determine the need of referral to a rheumatologist [9]. Prompt identification and therapy of patients with Pso at risk to develop PsA is crucial because treatment response after clinical manifestation of PsA is poor [11].

A higher prevalence of PsA has been observed in patients with severe Pso [17]. The mean PASI of the patients included in this study was more than 10, indicating a moderate to severe Pso, which was expected in these dermatology settings. A fast reduction of PASI was observed within 3 months after initiation of the therapy. Because the patients were treated with three different TNF-α inhibitors, including etanercept with slower onset and limited efficacy on Pso, infliximab with fast results after intravenous administration, and adalimumab as gold standard anti–TNF-α therapy for skin and joint involvement [18,19,20], the PASI rates of the cohort might reflect an average of these three therapies. However, the improvement does not achieve the expected efficacy of newer classes of biologics, such as interleukin (IL)-23 inhibitors [21].

Similarly, nail psoriasis significantly improved within the first three months of treatment, reaching a NAPSI score of approximately 23 and further improved throughout the entire observation time. A recently published study showed similar efficacy on nail psoriasis in patients with or without PsA treated with adalimumab [22], supporting that TNF-α inhibitors are an efficacious treatment option for nail psoriasis independently of the presence of PsA.

Considered one of the most promising treatments for PsA, TNF-α inhibitors are strongly recommended as the first treatment option if conventional disease modifying drugs are unsuccessful [23]. In this observational study, TNF-α inhibitors were introduced as second-line therapy in patients with Pso with nail and joint involvement. Switch of therapy was mainly based on dermatologic criteria, which could explain the initially moderate PsA activity of the included patients in terms of tender and swollen joints. 

Despite having only moderate PsA activity initially, patients showed significant responses to therapy with TNF-α inhibitors during the observational period. According to swollen joint status, low disease activity of PsA was reached after 24 months (mean 76-SJC of 1.8), which is in accordance with current recommendations for the management in PsA [24,25]. A limitation is that for therapy monitoring of PsA, not all parameters for the calculation of minimal disease activity score were considered because this score was not established at the study’s onset. However, additional criteria, such as the patient pain visual analog scale (VAS) or the patient global activity VAS, could easily be added to the common questionnaires that patients with PsA receive in the dermatology practice. These additional questions would encourage dermatologists to follow up with patients on disease activity and effectiveness of the PsA treatment in an independent and professional manner in case collaboration with a rheumatologist is not possible.

In this analysis, there was no correlation between NAPSI and affected DIP joint, which was in contrast to previous findings in the cross-sectional study by Lai et al. who found that a significant proportion of patients with radiologically diagnosed PsA had concomitant nail involvement and DIP arthritis [26]. In our study, patients were clinically examined and pre-selected as candidates for systemic treatment with anti-TNFα. As expected, the patients’ QoL improved with therapy even after 2 years of continuous therapy. However, the QoL of the patients was still affected, as implied by a mean DLQI score of 4.6 ± 5.7 in Month 24. In a recently published study, DLQI score in patients with PsA was shown to be higher than in patients with psoriasis but without arthritis [27]. An HAQ score below 0.5, as a patient-reported outcome for disability, has been established as a functional target for disease control in rheumatoid arthritis [28], and it is generally accepted as a cutoff of intact ability in other diseases. In this study, patients with PsA treated with a TNF-α inhibitor reached an HAQ score below this limit 3 months after initiation of treatment. The reduction of 0.3 units, the clinically significant decrease [29], was achieved after 9 months of treatment. Patients with PsA report significantly lower scores regarding physical functioning, pain, role limitations, and general health perceptions compared with the general population [30]. TNF-α inhibitors could be a promising therapy for patients with PsA to quickly regain lost functionality.

The scope of this study was to evaluate the effectiveness of TNF-α inhibitors in the real-world dermatology practice, where some dermatologists may lack the expertise to precisely evaluate the disease activity in PsA. To address this deficiency, recruiting sites were advised to keep the same examiner for each patient throughout the observational time to avoid between-rater discrepancies. Additionally, self-explained questionnaires with visualization of PsA examination were provided. Regrettably, enthesitis has not been examined in this study.

Undoubtedly, blocking TNF-α remains a promising target for the treatment of plaque type or nail psoriasis and PsA. In addition, in the interdisciplinary approach of coexisting inflammatory diseases, such as inflammatory bowel disease, hidradenitis suppurativa, or arthritis, TNF-α inhibitors still have a prominent position [31,32]. However, the evolution of new targets for biologics, such as IL-17 or IL-23, or small molecules targeting intracellular signals, have recently revolutionized the spectrum and goals of psoriasis treatment strategies [1,33]. New antibodies against IL-17 or IL-23 have been proven to be effective in PsA, or even axial Spondyloarthritis in the case of anti-IL-17, with a convenient injection schema and a favorable safety profile [25]. Similar results have been observed in the efficacy of IL-17 and IL-23 antibodies in the treatment of nail psoriasis. Particularly in the long term, the efficacy of all these three classes of biologics, anti-TNFα, anti-IL-17 and anti-IL-23, on nail psoriasis does not seem to differ significantly [34]. A personalized approach should be considered in Pso patients with PsA and nail involvement.

## 5. Conclusions

TNF-α inhibitors are an efficacious therapy for disease control and improvement of QoL in psoriasis patients with nail psoriasis and concomitant PsA. Dermatologists should regularly monitor psoriasis patients at risk of developing PsA and promptly initiate the appropriate therapy.

## Figures and Tables

**Table 1:** Demographic and clinical characteristics of patients at baseline.

Characteristic	Value
Age, years, mean ± SD	49.6 ± 12.4
Sex, %	
Female	37.0
Male	63.0
Disease duration, years, mean ± SD	20.1 ± 13.7
BMI, kg/m^2^, mean ± SD	28.7 ± 5.5
BSA at baseline, %, mean ± SD	27.5 ± 25.3
PASI at baseline, mean ± SD	15.0 ± 12.5
NAPSI at baseline, mean ± SD	33.8 ± 21.4
Tender joints at baseline, n, mean ± SD	10.8 ± 11.5
Swollen joints at baseline, n, mean ± SD	6.4 ± 9.5
Morning stiffness, %	
Yes	41.0
No	28.0
Unknown	31.0
DLQI at baseline, mean ± SD	12.5 ± 7.4
HAQ at baseline, mean ± SD	0.63 ± 0.61

BMI: body mass index; BSA: body surface area; DLQI: Dermatological Life Quality Index; HAQ: Health Assessment Questionnaire; NAPSI: Nail Psoriasis Severity Index; PASI: Psoriasis Area and Severity Index; SD: standard deviation.

## Data Availability

The data presented in this manuscript are available on request from the corresponding author. The data are not publicly available since not all data have been published yet.
